# Progressive Macroglossia and Dysphagia as the Initial Manifestation of Systemic Amyloid Light-Chain (AL) Amyloidosis Associated With Multiple Myeloma: A Case Report and Literature Review

**DOI:** 10.7759/cureus.108908

**Published:** 2026-05-15

**Authors:** Navanita Biswas, Motaz Almahmood, Shobha Mandal, Rashmika Potdar, Jian J Fu

**Affiliations:** 1 Internal Medicine, Reading Hospital, Tower Health, West Reading, USA; 2 Internal Medicine, Tower Health Medical Group, Phoenixville, USA; 3 Internal Medicine, Guthrie Robert Packer Hospital, Sayre, USA; 4 Hematology and Oncology, Tower Health Medical Group, Phoenixville, USA; 5 Pathology, Reading Hospital, Tower Health, West Reading, USA

**Keywords:** al-amyloidosis, bortezomib, dysphagia, macroglossia, multiple myeloma

## Abstract

Systemic amyloid light-chain (AL) amyloidosis is characterized by the extracellular deposition of misfolded immunoglobulin light chains in tissues and is often associated with plasma cell dyscrasias, including multiple myeloma. Macroglossia is an uncommon but characteristic manifestation and may rarely be the presenting feature. We report the case of a 68-year-old woman who presented with progressive tongue enlargement, dysphagia, and significant weight loss over six months. Laboratory evaluation revealed severe anemia, hypercalcemia, markedly elevated serum free kappa light chains with a profoundly abnormal kappa/lambda ratio, and an IgG kappa monoclonal protein. Because the degree of macroglossia raised concern for coexisting amyloidosis, an abdominal fat pad biopsy was performed, but it was negative. Targeted tongue biopsy subsequently demonstrated amyloid deposition, and bone marrow biopsy revealed extensive plasma cell infiltration consistent with multiple myeloma. The patient was treated with a bortezomib-based regimen (CyBorD), with daratumumab added subsequently, resulting in improvement in macroglossia, oral intake, weight, and hematologic parameters over six months. This case highlights progressive macroglossia as an important clinical clue to underlying systemic AL amyloidosis and plasma cell dyscrasia, and underscores the importance of early tissue diagnosis and prompt therapy.

## Introduction

Systemic amyloidosis is a heterogeneous group of disorders characterized by extracellular deposition of misfolded protein fibrils within tissues and organs, leading to progressive organ dysfunction. The most common type, amyloid light-chain (AL) amyloidosis, results from deposition of monoclonal immunoglobulin light chains produced by a clonal plasma cell population and is frequently associated with plasma cell dyscrasias such as multiple myeloma [1,2]. Clinical manifestations are variable and depend on the organs involved, with the kidneys, heart, liver, gastrointestinal tract, and peripheral nervous system among the most commonly affected sites [2,3].

Macroglossia is an uncommon but characteristic manifestation of systemic AL amyloidosis and results from amyloid deposition within the tongue musculature and surrounding soft tissues. It may lead to progressive tongue enlargement, dysphagia, impaired speech, and, in severe cases, airway compromise. Although macroglossia is considered a distinctive clinical feature of amyloidosis, it rarely serves as the initial presenting manifestation leading to the diagnosis of an underlying plasma cell disorder [3-5].

Here, we report a case of progressive macroglossia caused by biopsy-proven amyloid deposition in the tongue, which ultimately led to the diagnosis of systemic AL amyloidosis associated with multiple myeloma. This case highlights the importance of recognizing unusual oral manifestations as potential early indicators of systemic disease and provides a focused review of previously reported cases presenting with macroglossia and/or dysphagia. Importantly, this case also illustrates that a negative surrogate tissue biopsy should not preclude direct sampling of the clinically involved organ when clinical suspicion remains high, and that biopsy of the most clinically affected site, in this case the tongue, may be necessary to establish the diagnosis.

## Case presentation

A 68-year-old woman with a past medical history of hypertension, hyperlipidemia, coronary artery disease status post-remote percutaneous coronary intervention on clopidogrel, asthma, anxiety, or depression presented to the emergency department with progressively worsening tongue swelling over six months. The patient reported associated dysphagia to both solids and liquids (initially to solids, later progressing to liquids), generalized weakness, and significant unintentional weight loss of approximately 50 pounds during the same period. She denied fever, nausea, vomiting, rash, or recent infections. Family history was notable for a brother with colon cancer, but there was no known family history of amyloidosis or plasma-cell dyscrasia. She was a former long-term cigarette smoker. Notably, she had previously been evaluated by otolaryngology and other outpatient physicians for her progressive tongue enlargement and dysphagia before this presentation. Still, a unifying diagnosis had not yet been established. This prolonged evaluation by multiple physicians highlights how tongue-predominant AL amyloidosis may initially be underrecognized and how diagnosis may be delayed. 

On presentation, the patient was hemodynamically stable but clinically ill-appearing, with marked enlargement of the tongue consistent with macroglossia (Figure 1). The initial laboratory evaluation revealed significant anemia with a hemoglobin level of 7.6 g/dL and hypercalcemia with a calcium level of 12.3 mg/dL. Computed tomography (CT) of the soft tissues of the neck did not demonstrate a focal neck mass or pathologic cervical lymphadenopathy. However, contrast-enhanced CT of the chest, abdomen, and pelvis revealed a 1.1 cm lytic lesion involving the manubrium (Figure 2). Electrocardiography showed low-voltage QRS complexes, raising concern for a possible infiltrative cardiomyopathy (Figure 3). Nuclear medicine bone scan showed uptake at the sternomanubrial junction corresponding to the lytic lesion, with additional diffuse patchy uptake throughout the osseous structures (Figure 4).

**Figure 1:**
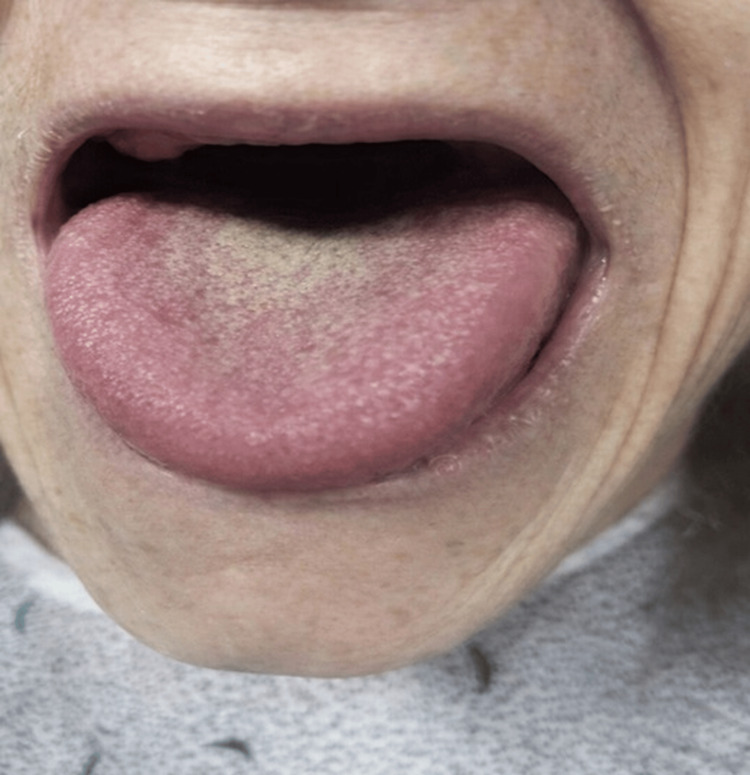
Clinical photograph demonstrating macroglossia Photograph obtained at presentation showing diffuse tongue enlargement consistent with macroglossia, which contributed to dysphagia and impaired oral intake

**Figure 2:**
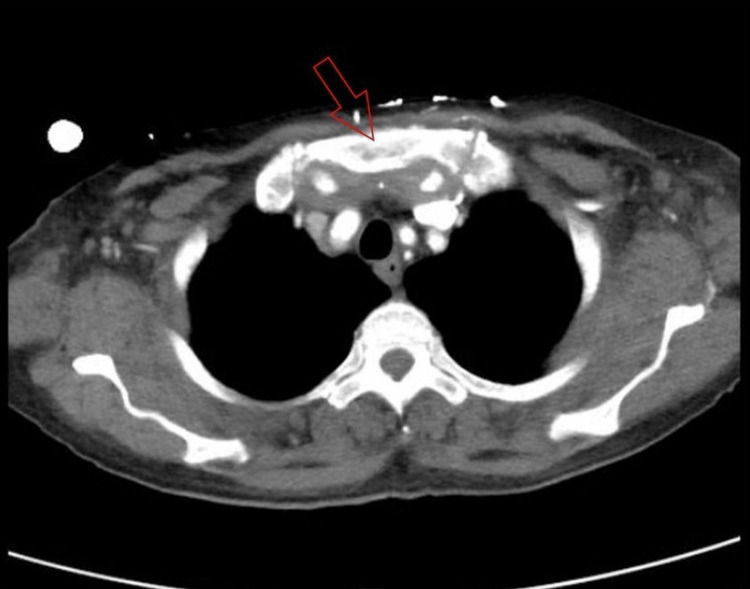
Computed tomography showing lytic bone lesion Contrast-enhanced computed tomography (CT) scan demonstrating a lytic lesion involving the manubrium, consistent with skeletal involvement in multiple myeloma

**Figure 3:**
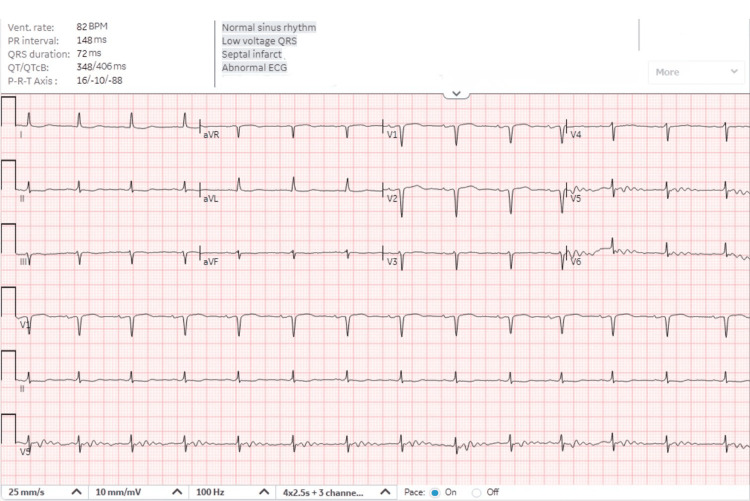
Electrocardiogram showing low-voltage QRS complexes Electrocardiographic findings demonstrated low-voltage QRS complexes, which may be seen with infiltrative cardiomyopathy including cardiac amyloid involvement

**Figure 4:**
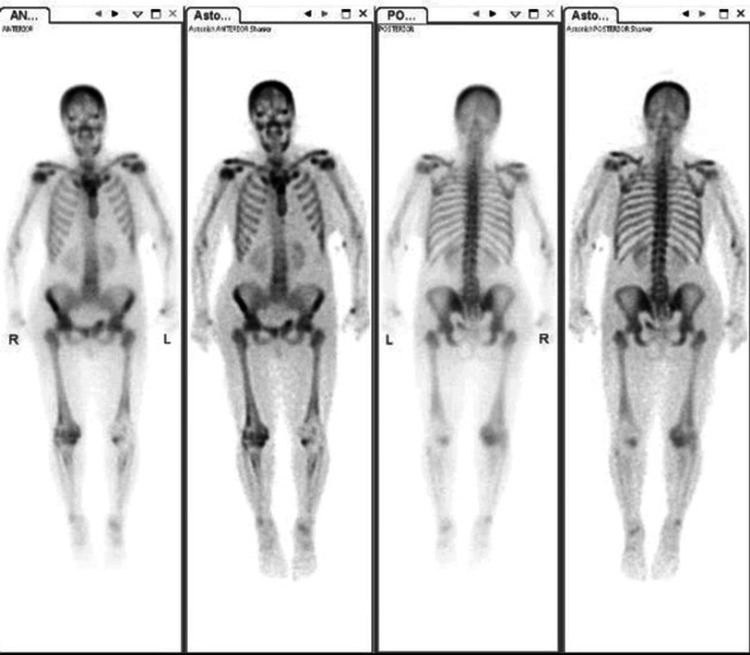
Nuclear medicine bone scan demonstrating sternomanubrial and diffuse osseous uptake Nuclear medicine bone scan shows focal uptake at the sternomanubrial junction corresponding to the lytic lesion, with additional diffuse patchy uptake throughout the osseous structures

Given the progressive macroglossia, otolaryngology was consulted. Flexible nasopharyngoscopy demonstrated edema of the arytenoids and postcricoid region with mild-to-moderate bilateral vocal cord edema. Gastroenterology was also consulted for evaluation of dysphagia; however, esophagogastroduodenoscopy was deferred because of high anesthetic risk in the setting of severe tongue enlargement and airway concerns. A videofluoroscopic swallow study demonstrated impaired swallowing mechanics, and the patient was subsequently placed on a pureed diet.

Further hematologic evaluation demonstrated markedly elevated serum free light chains, with a kappa level of 11,970 mg/L and a lambda level of 2.5 mg/L, resulting in a markedly abnormal kappa/lambda ratio. Serum protein electrophoresis demonstrated a monoclonal (M) spike of 0.5 g/dL, and immunofixation electrophoresis confirmed an IgG kappa monoclonal gammopathy. Serum beta-2 microglobulin was elevated at 11.9 mg/L, consistent with International Staging System (ISS) stage III multiple myeloma (laboratory results are summarized in Tables 1-6).

**Table 1:** Complete blood count at time of presentation

Parameter	Patient value	Reference range	Units
White blood cells	5.2	4.8-10.8	×10³/μL
Red blood cells	2.41	4.2-5.4	×10⁶/μL
Hemoglobin	7.6	12.0-16.00	g/dL
Hematocrit	23.6	36-46	%
Mean corpuscular volume (MCV)	97.9	80-99	fL
Mean corpuscular hemoglobin (MCH)	31.5	27 – 34	pg
Mean corpuscular hemoglobin concentration (MCHC)	32.2	31-37	g/dL
Red cell distribution width (RDW)	17.9	11-16	%
Platelets	185	130 – 400	×10³/μL

**Table 2:** Comprehensive metabolic panel AST: aspartate aminotransferase; SGOT: serum glutamic-oxaloacetic transaminase; ALT: alanine aminotransferase; SGPT: serum glutamate pyruvate transaminase

Test	Result	Reference range	Units
Sodium	139	135-145	mmol/L
Potassium	3.9	3.5-5.0	mmol/L
Chloride	101	98-106	mmol/L
Bicarbonate (HCO3)	26	22-29	mmol/L
Anion gap	12	8-16	mmol/L
Blood urea nitrogen (BUN)	20	7-20	mg/dL
Creatinine	0.91	0.6-1.3	mg/dL
Glucose	93	70-99	mg/dL
Calcium	12.3	8.5-10.5	mg/dL
AST (SGOT)	21	10-40	U/L
ALT (SGPT)	8	7-56	U/L
Direct bilirubin	0.1	0.0-0.3	mg/dL
Total bilirubin	0.5	0.1-1.2	mg/dL
Alkaline phosphatase	144	46-114	U/L
Albumin	3.7	3.4-5.0	g/dL
Total protein	7.4	5.7-8.2	g/dL

**Table 3:** Urine chemistry findings

Test	Result	Reference range
24-hour urine total protein	4070 mg/24 h	<150 mg/24 h
Urine total protein/creatinine ratio	5952 mg/g creatinine	24-184 mg/g creatinine

**Table 4:** 24-hour urine monoclonal protein evaluation

Test	Result	Reference range
24-hour urine protein electrophoresis (UPEP)	Abnormal monoclonal protein band detected	None detected
Monoclonal protein band (Band 1)	387 mg/dL	None detected
Urine immunofixation electrophoresis	IgG kappa monoclonal protein detected	None detected

**Table 5:** Serum monoclonal protein studies

Test	Result	Reference range
Serum protein electrophoresis (SPEP)	Faint restricted band (M-spike) in the gamma globulin region	No monoclonal protein detected
Serum immunofixation electrophoresis	IgG kappa monoclonal band present	No monoclonal band detected
Serum free kappa light chains	11,970 mg/L	3.3-19.4 mg/L
Serum free lambda light chains	2.5 mg/L	5.7-26.3 mg/L
Kappa/lambda free light chain ratio	583.67	0.26-1.65

**Table 6:** Additional laboratory studies

Test	Result	Reference range
Thyroid-stimulating hormone (TSH)	10.049 µIU/mL	0.550-4.780 µIU/mL
Free thyroxine (free T4)	1.04 ng/dL	0.89-1.76 ng/dL
Vitamin B12	312 pg/mL	211-911 pg/mL

Abdominal fat pad biopsy was negative for amyloid deposition (Figure 5). A biopsy of the left oral tongue was performed to evaluate the persistent macroglossia. Histopathologic examination demonstrated squamous mucosa with amyloid deposition and epithelial hyperplasia (Figure 6). Congo red staining confirmed amyloid deposition within the lamina propria, focally involving vascular walls and skeletal muscle fibers (Figure 7) and demonstrated characteristic birefringence under polarized light (Figure 8), confirming the presence of amyloid. Mass spectrometry further identified the amyloid as an AL (kappa) subtype. Bone marrow biopsy demonstrated a hypercellular marrow with approximately 65% cellularity and 80% plasma cells (Figure 9). Plasma cells were kappa-restricted by in situ hybridization and expressed CD38 and CD138 (Figure 10) with CD19 negativity and CD56 positivity. Cytogenetic analysis revealed deletion 13q, deletion 14q, and trisomies of chromosomes 5, 9, and 15. Taken together, the findings were most consistent with systemic AL amyloidosis associated with IgG kappa multiple myeloma. Myeloma-defining events included anemia, hypercalcemia, and a lytic bone lesion.

**Figure 5:**
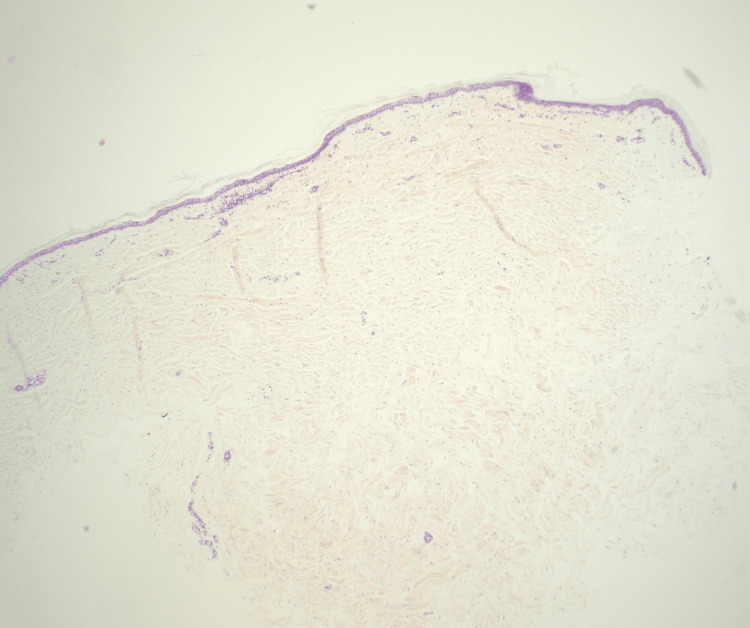
Abdominal fat pad biopsy with no definite amyloid deposition on Congo red stain Low-power Congo red-stained section of abdominal fat pad biopsy showing no convincing amyloid deposition, consistent with a negative surrogate tissue study

**Figure 6:**
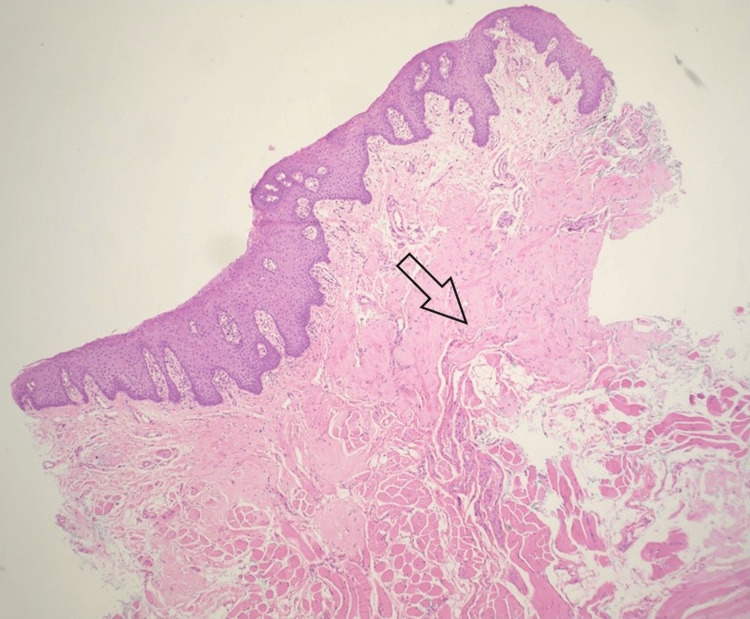
Tongue biopsy demonstrating subepithelial amyloid deposition Histopathologic section of the tongue shows squamous mucosa with epithelial hyperplasia and amyloid deposition within the lamina propria (arrow)

**Figure 7:**
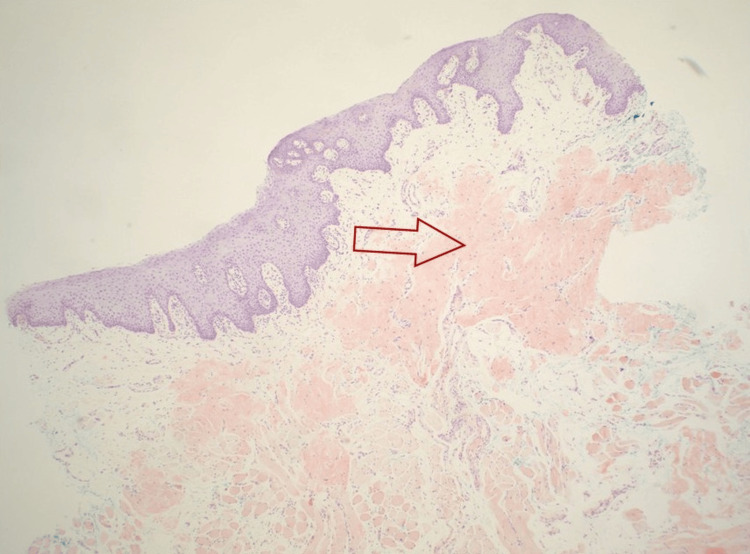
Tongue biopsy demonstrating amyloid deposition on Congo red stain Low-power histopathologic section of the tongue showing squamous mucosa overlying Congo red-positive amyloid deposition in the lamina propria and underlying soft tissue

**Figure 8:**
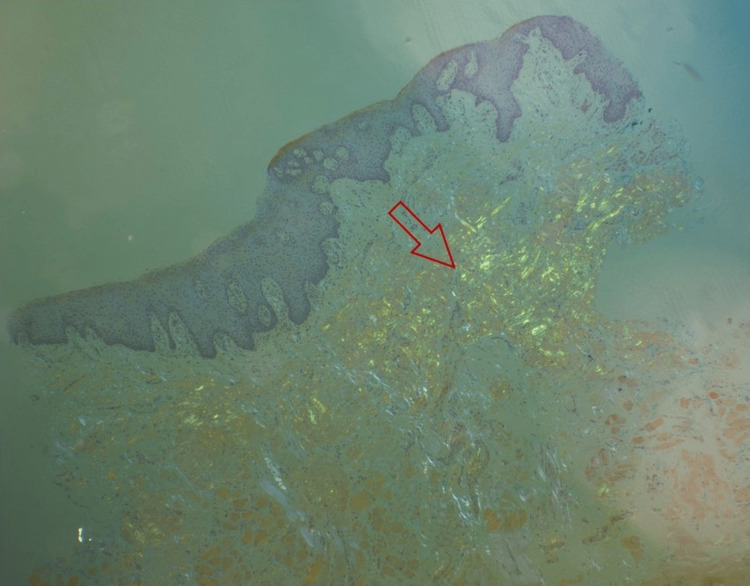
Tongue biopsy showing apple-green birefringence under polarized light Congo red-stained section of the tongue demonstrates apple-green birefringence consistent with amyloid deposition (arrow) under polarized light

**Figure 9:**
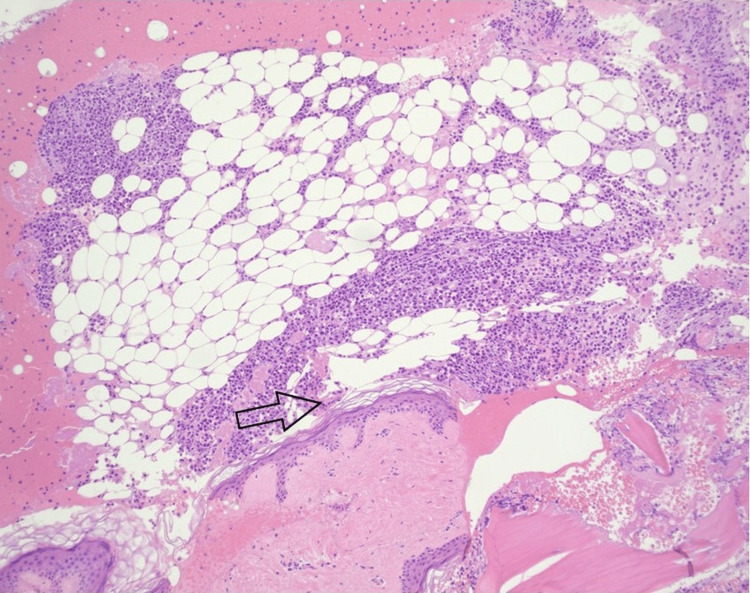
Bone marrow biopsy demonstrating marked plasma cell infiltration Histopathologic examination of the bone marrow shows a hypercellular marrow with extensive plasma cell infiltration (arrow), consistent with plasma cell neoplasm and supporting the diagnosis of multiple myeloma

**Figure 10:**
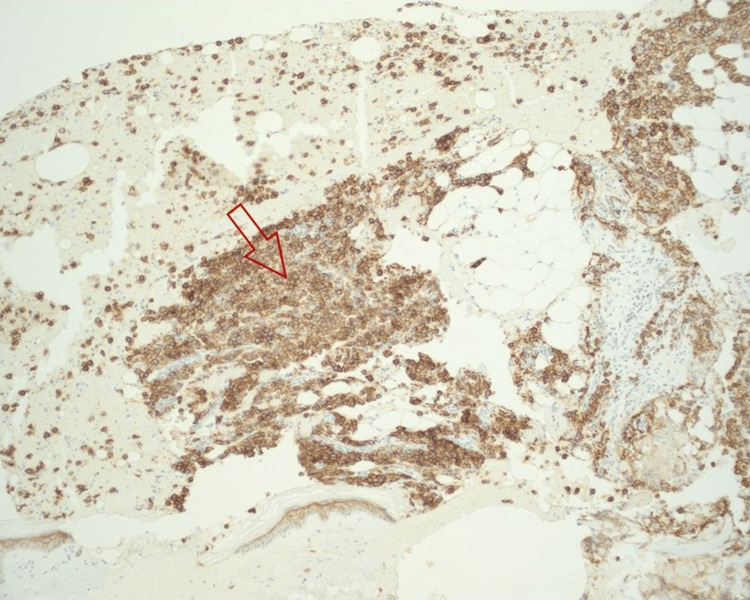
Bone marrow biopsy with diffuse CD138-positive plasma cells CD138 immunohistochemistry highlights extensive plasma cell infiltration of the bone marrow, consistent with multiple myeloma in the appropriate clinical and immunophenotypic context

Cardiac evaluation was performed to assess for possible cardiac involvement. N-terminal pro-B-type natriuretic peptide (NT-proBNP) was elevated at 577 pg/mL. Transthoracic echocardiography demonstrated normal left ventricular size, wall thickness, and systolic function without regional wall motion abnormalities. Left ventricular ejection fraction was 67%, while global longitudinal strain was mildly reduced at -12.9%. The right ventricle was normal in size and function. These findings raised concern for possible early cardiac involvement, although overt structural cardiac amyloidosis was not definitively established. Cardiac MRI was not performed during the index hospitalization due to the prioritization of urgent hematologic workup and treatment initiation; this represents a limitation of the present case, as cardiac MRI would have provided more definitive characterization of myocardial involvement.

The patient was initiated on combination chemotherapy with bortezomib, cyclophosphamide, and dexamethasone (CyBorD) and received intravenous fluids, with eventual resolution of hypercalcemia. Early outpatient testing already showed a reduction in hematologic burden, with kappa free light chains declining to 1,751 mg/L, lambda free light chains of 3.0 mg/L, a kappa/lambda ratio of 583.7, and a faint residual IgG kappa M-spike of approximately 0.4 g/dL. Daratumumab was subsequently added to CyBorD in the outpatient setting. Lenalidomide was not initiated because of significant proteinuria concerning for nephrotic-range renal involvement and its potential to worsen renal dysfunction. A 24-hour urine collection demonstrated approximately 980 mg/24 hours of proteinuria with a monoclonal free kappa band and M-protein in the alpha-2 region. Follow-up random urine protein electrophoresis with immunofixation remained abnormal, showing urine protein of 97.8 mg/dL, albumin of 12.8%, alpha-1 globulin of 2.0%, alpha-2 globulin of 67.5%, beta globulin of 12.9%, gamma globulin of 4.7%, and a urine M-spike of 60.6%; immunofixation demonstrated an IgG monoclonal protein with kappa light-chain specificity together with monoclonal free kappa light chains (Bence Jones protein). 

Across serial outpatient visits over approximately six months, both clinical and hematologic parameters improved. Hemoglobin stabilized in the 9-10 g/dL range. Macroglossia gradually diminished, oral intake improved, and body weight rose from approximately 50 kg to approximately 58-61 kg. Serum kappa free light chains fell sequentially from 11,970 mg/L at diagnosis to 364 mg/L. A 24-hour urine collection at baseline demonstrated approximately 4,070 mg/24 hours of proteinuria prior to treatment. Early outpatient follow-up after treatment initiation showed improvement to approximately 980 mg/24 hours, and by approximately six months, 24-hour urine protein had further decreased to 123 mg/24 hours. Transplant consultation considered her a borderline candidate for stem cell transplantation pending further cardiac and renal staging, and transplantation was deferred while she continued to deepen her response on daratumumab-CyBorD. The stepwise clinical course is summarized in Figure 11.

**Figure 11:**
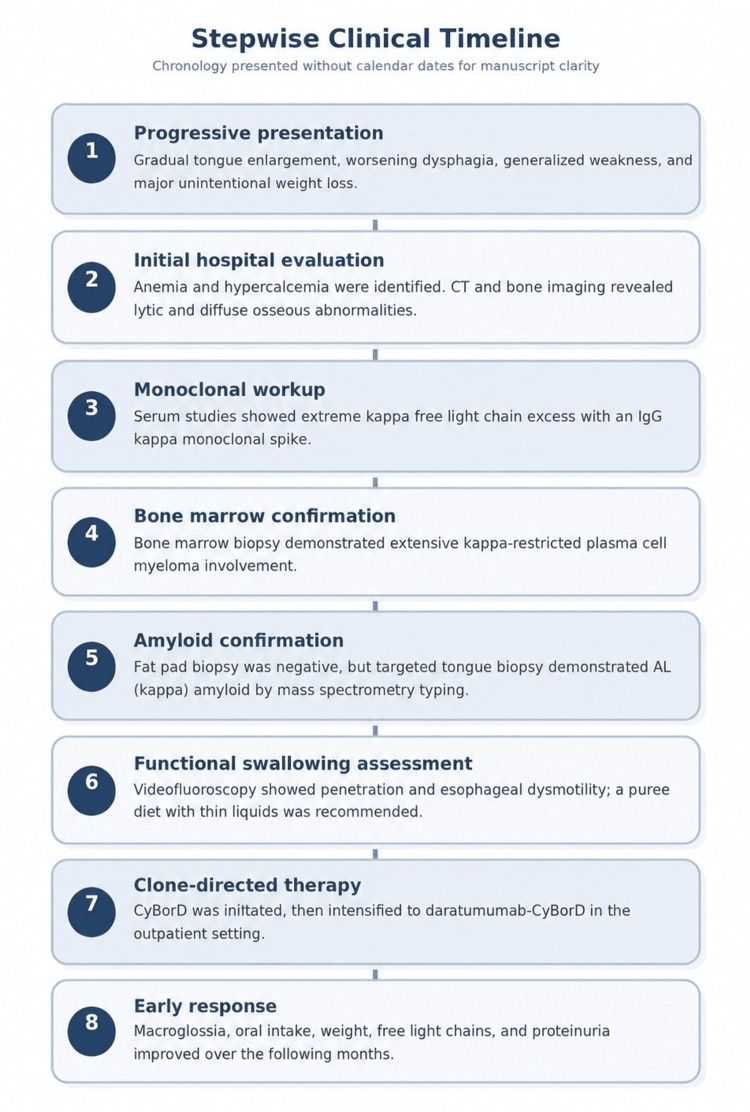
Stepwise, date-free clinical timeline from presenting symptoms through early hematologic and clinical response Timeline summarizing the patient’s presenting symptoms, diagnostic workup, treatment course, and early clinical and hematologic response. This figure was created using Microsoft PowerPoint

## Discussion

Macroglossia is an uncommon clinical finding characterized by persistent enlargement of the tongue and may lead to dysphagia, dysarthria, airway compromise, and impaired oral intake [6]. Its etiology can be broadly categorized into congenital and acquired causes. Congenital causes include Down syndrome, Beckwith-Wiedemann syndrome, and vascular malformations, whereas acquired causes include endocrine disorders such as hypothyroidism, inflammatory conditions, neoplasms, and infiltrative disorders such as amyloidosis [6,7]. Among these, amyloidosis remains one of the most recognized causes of progressive macroglossia in adults.

Amyloidosis refers to a group of disorders characterized by extracellular deposition of misfolded protein fibrils in various tissues and organs. Major subtypes include AL amyloidosis, amyloid A (AA) amyloidosis associated with chronic inflammatory conditions, hereditary amyloidosis, and transthyretin amyloidosis [1]. Among these, AL amyloidosis is the subtype most commonly associated with plasma cell dyscrasias. It results from deposition of monoclonal immunoglobulin light chains produced by abnormal plasma cells, leading to progressive organ dysfunction. The disease typically presents in the sixth to seventh decades of life and commonly involves the kidneys, heart, liver, gastrointestinal tract, and peripheral nerves [2].

Macroglossia is considered a classic but relatively uncommon manifestation of AL amyloidosis, occurring in approximately 10%-20% of cases [3]. AL amyloid infiltration of the tongue leads to progressive enlargement and may be associated with scalloping of the lateral tongue margins, speech impairment, dysphagia, and nutritional compromise. Because macroglossia is unusual and often develops gradually, diagnosis may be delayed unless AL amyloidosis is specifically considered.

The diagnostic evaluation of suspected amyloidosis requires histopathologic confirmation of amyloid deposition. Tissue biopsy with Congo red staining demonstrating apple-green birefringence under polarized light remains the gold standard for diagnosis. Common biopsy sites include abdominal fat pad aspirate, bone marrow, or clinically involved organs. However, the sensitivity of abdominal fat pad biopsy is variable. In one study of patients with cardiac amyloidosis, amyloid was detected in 84% of those with AL amyloidosis, with lower sensitivity in patients with smaller whole-body amyloid burden [8]. Therefore, biopsy of involved tissue may be required when initial testing is negative. In the present case, abdominal fat pad biopsy was negative, whereas tongue biopsy confirmed amyloid deposition involving the lamina propria, vascular walls, and skeletal muscle fibers. This underscores the importance of targeted tissue sampling when clinical suspicion remains high.

AL amyloidosis is frequently associated with an underlying plasma cell dyscrasia, most commonly multiple myeloma or monoclonal gammopathy of undetermined significance. Approximately 10%-15% of patients with AL amyloidosis have concurrent multiple myeloma [9,10]. In our patient, serum protein electrophoresis demonstrated a monoclonal spike, immunofixation confirmed an IgG kappa monoclonal protein, and serum free light chain analysis revealed markedly elevated kappa light chains with a profoundly abnormal kappa/lambda ratio. Bone marrow biopsy further demonstrated extensive plasma cell infiltration, confirming the presence of multiple myeloma.

Evaluation of AL amyloidosis should also include assessment for additional organ involvement, particularly the heart and kidneys, because multiorgan involvement strongly influences prognosis. Cardiac involvement is a major determinant of survival and is commonly evaluated using cardiac biomarkers and echocardiography. In this case, elevated NT-proBNP and mildly reduced global longitudinal strain raised concern for possible early cardiac involvement, although left ventricular systolic function remained preserved.

Treatment of AL amyloidosis associated with multiple myeloma is directed at suppression of the underlying plasma cell clone to reduce production of pathogenic light chains. Bortezomib-based therapy, including cyclophosphamide, bortezomib, and dexamethasone (CyBorD), is a well-established treatment approach in this setting [11,12]. More recently, daratumumab-containing regimens have also become an important component of therapy for AL amyloidosis [13]. In our patient, CyBorD was initiated during hospitalization, and daratumumab was subsequently added in the outpatient setting. On subsequent follow-up, she had marked clinical improvement with a reduction in tongue swelling, improved speech articulation, and better tolerance of oral intake. Anemia and hypercalcemia also improved, and serial serum free light chain measurements demonstrated a downward trend, consistent with treatment response.

Literature review and comparison with published cases

To contextualize the present case, a focused, nonsystematic search of PubMed and Google Scholar was undertaken for articles published through April 2026 using combinations of the terms "AL amyloidosis," "multiple myeloma," "macroglossia," "dysphagia," "tongue," and "oral amyloidosis," with additional hand-searching of reference lists. Only reports in which macroglossia and/or dysphagia were an initial or early presenting manifestation that led to the diagnosis of AL amyloidosis, multiple myeloma, or both were retained for comparison [14-21]. Broader oral amyloidosis series, previously known myeloma cohorts, and reports without an initial macroglossia/dysphagia phenotype were not tabulated. Key features of comparable published cases are summarized in Table 7.

**Table 7:** Comparable published case reports of AL amyloidosis with concurrent or newly recognized multiple myeloma presenting initially with macroglossia and/or dysphagia AL: amyloid light-chain; MM: multiple myeloma

Study	Initial presenting manifestation	Confirmation of AL amyloidosis/MM	Relevance to the present case
da Costa et al., 2018 [14]	Dysphagia due to macroglossia	Oral/tongue histopathology confirmed amyloidosis; hematologic evaluation established associated MM	Closest symptom match, with dysphagia and tongue enlargement as the dominant presenting complaints
Dash et al., 2024 [15]	Hoarseness and dysphagia, followed by tongue thickening/macroglossia	Tongue and esophageal biopsies showed AL; bone marrow showed >30% kappa-restricted plasma cells	Very close overlap in upper aerodigestive symptoms, tongue involvement, and kappa-restricted plasma-cell disease
Brandstetter et al., 2012 [16]	Macroglossia with bilateral submandibular swelling	Tongue biopsy established amyloidosis; serum immunofixation identified kappa light-chain gammopathy from MM	Strong tongue-first comparator because the involved-site tongue biopsy was decisive, similar to the present case
Finsterer et al., 1997 [17]	Dysarthria, mild tongue enlargement, and dysphagia	Tongue biopsy showed amyloid; bone marrow biopsy revealed light-chain myeloma	Strong functional analogue in which swallowing symptoms and tongue amyloid preceded recognition of the plasma-cell disorder
Cherico et al., 2022 [18]	Isolated worsening macroglossia	Tongue biopsy with Congo red confirmed AL; bone marrow biopsy confirmed concurrent MM	Similar tongue-first presentation, although dysphagia was absent, and cardiac involvement was more advanced
Maturana-Ramirez et al., 2018 [19]	Macroglossia with weight loss	Tongue amyloidosis was diagnosed first; systemic evaluation then disclosed MM	Supports the tongue-first diagnostic pathway, although dysphagia was less prominent than in the present case
Demirkan et al., 2017 [20]	Tongue enlargement as the first clinical sign	Kidney and bone marrow biopsies were Congo red-positive for AL; marrow showed 30% plasma cells	Similar macroglossia-first presentation, but confirmatory tissue was obtained outside the tongue
Xue et al., 2024 [21]	Progressive dysphagia as the first symptom	Biceps pathology indicated AL; bone marrow/pathology confirmed MM	Useful dysphagia-first comparator, but macroglossia and tongue-dominant disease were not central features.

The closest comparators are da Costa et al. and Dash et al., because both reports combined dysphagia with tongue enlargement or macroglossia at presentation and both ultimately demonstrated AL amyloidosis associated with multiple myeloma [14-15]. Brandstetter et al., Finsterer et al., Cherico et al., and Maturana-Ramirez et al. further support a tongue-first or bulbar-first phenotype in which macroglossia, tongue enlargement, or swallowing symptoms triggered tissue diagnosis and subsequent hematologic evaluation [16-19]. Demirkan et al. and Xue et al. remain relevant but are slightly less direct comparators because amyloid confirmation depended on non-tongue tissue in one case and tongue-dominant disease was less central in the other [20,21].

Taken together, these reports show that unexplained macroglossia and/or progressive dysphagia can be the earliest clinically recognizable manifestation of systemic AL amyloidosis and may be the clue that leads to recognition of an underlying plasma-cell disorder [14-21]. The present case fits this phenotype but adds an important diagnostic lesson: negative surrogate tissue studies should not exclude AL amyloidosis when clinical suspicion remains high. In our patient, abdominal fat pad aspiration and bone marrow Congo red staining were non-diagnostic, whereas biopsy of the clinically involved tongue established AL (kappa) amyloid and directed definitive diagnosis of concurrent multiple myeloma. This reinforces the value of early biopsy from the most clinically involved site when tongue-predominant disease is present.

## Conclusions

Macroglossia is a rare but important presenting manifestation of systemic AL amyloidosis and may be the first clue to an underlying plasma cell disorder such as multiple myeloma. Early recognition of progressive tongue enlargement, followed by timely tissue biopsy and hematologic evaluation, can facilitate prompt diagnosis and initiation of therapy. In the present case, bortezomib-based therapy followed by the addition of daratumumab was associated with meaningful improvement in both systemic disease burden and local soft tissue manifestations. This case underscores the importance of considering amyloidosis in adults with unexplained progressive macroglossia in order to avoid delayed diagnosis and optimize outcomes.
